# Contrasting extracellular enzyme activities of particle-associated bacteria from distinct provinces of the North Atlantic Ocean

**DOI:** 10.3389/fmicb.2012.00425

**Published:** 2012-12-13

**Authors:** Carol Arnosti, Bernhard M. Fuchs, Rudolf Amann, Uta Passow

**Affiliations:** ^1^Department of Marine Sciences, University of North Carolina at Chapel HillChapel Hill, NC, USA; ^2^Department of Molecular Ecology, Max Planck Institute for Marine MicrobiologyBremen, Germany; ^3^Alfred Wegener Institute for Polar and Marine ResearchBremerhaven, Germany

**Keywords:** extracellular enzymes, biogeography, particles-associated bacteria, hydrolysis, carbon cycling

## Abstract

Microbial communities play a key role in the marine carbon cycle, processing much of phytoplankton-derived organic matter. The composition of these communities varies by depth, season, and location in the ocean; the functional consequences of these compositional variations for the carbon cycle are only beginning to be explored. We measured the abilities of microbial communities in the large-particle fraction (retained by a 10-μm pore-size cartridge filter) to enzymatically hydrolyze high molecular weight substrates, and therefore initiate carbon remineralization in four distinct oceanic provinces: the boreal polar (BPLR), the Arctic oceanic (ARCT), the North Atlantic drift (NADR), and the North Atlantic subtropical (NAST) provinces. Since we expected the large-particle fraction to include phytoplankton cells, we measured the hydrolysis of polysaccharide substrates (laminarin, fucoidan, xylan, and chondroitin sulfate) expected to be associated with phytoplankton. Hydrolysis rates and patterns clustered into two groups, the BPLR/ARCT and the NADR/NAST. All four substrates were hydrolyzed by the BPLR/ARCT communities; hydrolysis rates of individual substrate varied by factors of ca. 1–4. In contrast, chondroitin was not hydrolyzed in the NADR/NAST, and hydrolytic activity was dominated by laminarinase. Fluorescence *in situ* hybridization of the large-particle fraction post-incubation showed a substantial contribution (15–26%) of CF319a-positive cells (*Bacteroidetes*) to total DAPI-stainable cells. Concurrent studies of microbial community composition and of fosmids from these same stations also demonstrated similarities between BPLR and ARCT stations, which were distinct from the NADR/NAST stations. Together, these data support a picture of compositionally as well as functionally distinct communities across these oceanic provinces.

## INTRODUCTION

Heterotrophic microbial communities collectively process a large fraction of the organic matter biosynthesized in the ocean (Azam, 1998), remineralizing, repackaging, and respiring a variety of substrates, and thus playing a central role in the marine carbon cycle. These communities have been shown recently to exhibit distinct biogeographic patterns in the ocean, with community composition differing by location, season, water mass, and depth (e.g., Kan et al., 2006; Agogue et al., 2011; Gilbert et al., 2012; Hanson et al., 2012). The consequences of these compositional differences for microbial community function are only beginning to be explored, however. The distribution of functional genes among communities at different locations, depths, and times in the ocean demonstrates the potential for distinct functionalities among these communities (DeLong et al., 2006; Teeling et al., 2012), but the conditions under which and extent to which potential differences in function might be expressed are still largely unknown. Although transcriptomic investigations have yielded insight into broad categories of genes that are active in marine microbial communities (Poretsky et al., 2009; Shi et al., 2011), this approach has major limitations with respect to carbon cycling, due to the wide range of potential substrates in ocean waters and our very limited abilities to identify specific functional genes related to cycling of these substrates (e.g., Rebuffet et al., 2011).

Direct measurements of carbon processing by microbial communities demonstrate their abilities to take up specific low molecular weight substrates labeled with ^14^C or ^3^H (e.g., Rich et al., 1997; Vila-Costa et al., 2007; Alonso-Saez et al., 2008), but these studies yield only indirect information about the rate at which most natural marine organic matter is remineralized, since most marine organic matter is initially biosynthesized as macromolecules such as polysaccharides, proteins, and lipid complexes. Although marine macromolecules are present in much higher concentrations than low molecular weight organic matter, microbial access to these substrates requires hydrolysis by extracellular enzymes to produce substrates that can be transported across cell membranes for further processing (Arnosti, 2011). The activities of these enzymes thus initiate carbon cycling by heterotrophic microbial communities, and determine the types of organic matter that can serve as substrates.

Efforts to measure activities of microbial extracellular enzymes also – paradoxically – usually rely on a small number of low molecular weight substrate proxies. These commercially available proxies consist of a monomer such as glucose or leucine linked to a fluorophore whose fluorescence increases greatly upon hydrolysis of the monomer-fluorophore bond (Hoppe, 1983). This experimental approach has been used to compare potential hydrolysis rates at a wide range of depths and locations in the water column (e.g., Huston and Deming, 2002; Baltar et al., 2009; Piontek et al., 2011), and results have frequently been extrapolated to the degradation of carbohydrates and proteins in general (Christian and Karl, 1995; Fukuda et al., 2000). These substrate proxies, however, do not adequately mimic the three-dimensional structure of organic macromolecules in solution, so the relationship between hydrolysis rates measured with these proxies and the hydrolysis rates of their putative macromolecular counterparts are highly uncertain (Warren, 1996).

In order to measure the activities of enzymes responsible for hydrolysis of high molecular weight organic matter, alternative methods have been developed. These methods are intended also to detect the activities of endo-acting enzymes that cleave macromolecules mid-chain, an essential step in microbial degradation of macromolecular organic matter (Weiner et al., 2008; McBride et al., 2009). These approaches require synthesis of specific labeled substrates: fluorescently labeled polysaccharides and phytoplankton extracts (Arnosti, 1995, 2003; Arnosti et al., 2005b) or peptides (Pantoja et al., 1997, 2009; Pantoja and Lee, 1999), and chromatographic separation of the hydrolysis products, and thus requires considerably more work pre- and post-experiment. The additional effort is compensated for by the fact that these substrates can be used to investigate differences in hydrolysis rates and patterns for substrates with closely related structures, yielding new insight into the specific enzymatic capabilities of heterotrophic microbial communities in the water column and sediments. Using this approach, major functional differences among heterotrophic microbial communities at different depths and locations in the ocean have been identified (Arnosti et al., 2005a; Arnosti, 2008; Steen et al., 2012), showing that specific complements of enzyme activities are found not just for individual organisms, but among entire microbial communities. Moreover, these patterns of differences in enzyme activities have been shown to extend along large spatial gradients in the ocean (Arnosti et al., 2011), perhaps reflecting large-scale changes in microbial community composition along latitudinal gradients (Pommier et al., 2007; Fuhrman et al., 2008).

Given previous evidence of substantial differences in microbial communities in the North Atlantic (Schattenhofer et al., 2009), we tested whether organisms from distinct oceanic provinces within near-surface waters of the North Atlantic differed in their abilities to hydrolyze high molecular weight substrates. We focused in particular on a fraction of seawater enriched in large particles, since particle-associated heterotrophic communities are believed to be well-equipped to metabolize high molecular weight substrates, with enzymes that are sufficiently active so as to provide hydrolysate to the surrounding water column community as well as to the attached community (Smith et al., 1992; Simon et al., 2002; Grossart, 2010). At the conclusion of our incubation experiments, we carried out fluorescence *in situ* hybridization (FISH) staining to compare communities from the same station that had been incubated with different substrates, as well as communities obtained from different locations that were incubated with the same substrate. Concurrently conducted investigations of microbial community composition (Gomez-Pereira et al., 2010; Schattenhofer et al., 2011) and *Bacteroidetes*-associated fosmids (Gomez-Pereira et al., 2012) from the same locations provided us with the opportunity to examine the link between microbial community composition and function, and thus provided a larger context within which to interpret our measurements of microbial community activity. Together, these data provide new insight into the functional capabilities as well as the composition of microbial communities in distinct oceanic provinces of near-surface waters of the North Atlantic.

## MATERIALS AND METHODS

### SAMPLE COLLECTION AND WATER MASS IDENTIFICATION

Water was collected at a depth of 20 m using Niskin bottles mounted on a rosette equipped with a CTD at four stations (S3, S6, S12, and S19) during the VISION cruise of the R/V *Maria S. Merian* (September/October 2006). The stations represented distinct oceanic provinces on a N–S gradient along the 30°W meridian. As discussed in detail in Gomez-Pereira et al. (2010), water mass provinces were defined via satellite-derived parameters including Advanced Very High Resolution Radiometer (AVHRR), sea surface temperature and Sea-viewing Wide Field-of-view Sensor (SeaWiFS) water leaving radiance, according to Oliver and Irwin (2008). By these definitions, S3 was in the boreal polar (BPLR), S6 was within the Arctic oceanic (ARCT) province, S12 was within the North Atlantic drift (NADR), and S19 was within the North Atlantic subtropical (NAST) province (Gomez-Pereira et al., 2010).

### SAMPLE PREPARATION AND MEASUREMENT OF EXTRACELLULAR ENZYMATIC HYDROLYSIS

At each station, ca. 100 l of seawater (combined contents of five Niskin bottles) were passed through a stainless steel cartridge filter (Wolf Technik, Weil der Stadt, Germany), resulting in approximately 1 l retentate with particles larger than 10 μm (**Table 1**). The microbial community of the retentate is operationally defined as the large-particle-associated fraction; this fraction was used for incubation experiments. We note that this operationally defined fraction necessarily contained some bacteria present in the surrounding seawater, since the particles in the retentate were not removed from surrounding water. Microscopy of the retentate revealed that protists (diatoms, dinoflagellates, ciliates, and many others) and ample debris of unknown origin were collected. Since large-particle-associated bacteria concentrated from the euphotic zone may preferentially be associated with phytoplankton cells, which are typically carbohydrate-rich (Parsons et al., 1961), we focused on measuring the potential of the bacteria retained within this fraction to hydrolyze high molecular weight substrates that would likely be associated with phytoplankton. Such substrates would include carbohydrates exudates, carbohydrate-containing cellular components, and phytoplankton-derived transparent exopolymeric particles (TEP; Painter, 1983; Passow et al., 1994; Myklestad, 1995). Four different fluorescently labeled polysaccharides (laminarin, xylan, fucoidan, and chondroitin sulfate) were therefore used to measure the activity of extracellular enzymes, after the method of Arnosti (1995), 2003. The polysaccharides were purchased from Sigma or Fluka, labeled with fluoresceinamine (Sigma; Isomer II), and characterized as described in Arnosti (2003). Laminarin (β(1,3)-glucose), xylan (β(1,4) xylose), and fucoidan (sulfated fucose) are components of marine algae and phytoplankton (Parsons et al., 1961; Painter, 1983); fucoidan additionally has a chemical composition consistent with TEP (Zhou et al., 1998). Chondroitin sulfate is a marine-derived polysaccharide (a sulfated polymer of galactosamine and glucuronic acid (β-GlcA (1,3)-GalNAc (1,4)) that is rapidly hydrolyzed by heterotrophic microbial communities in seawater and sediments (Arnosti, 2008; Arnosti et al., 2009). Most of these polysaccharides are produced by marine phytoplankton and algae (Painter, 1983; Alderkamp et al., 2007). Moreover, activities of enzymes hydrolyzing all of these substrates have been measured in seawater and sediments (Arnosti et al., 2005a; Arnosti, 2008; Teske et al., 2011), and gene sequences corresponding to enzymes that would hydrolyze these substrate have also been identified in the genomes of recently sequenced marine bacteria (Glöckner et al., 2003; Bauer et al., 2006; Weiner et al., 2008).

**Table 1:** Station location, sample volume, and physical and chemical characteristics of the water column at 20 m depth.

**Station**	**Position**	***In situ* temperature (°C)**	Salinity (PSU)*	**Water mass**	**10 μm filter concentration**		**Concentration factor**	PO_4_^3−^ (μM)*	NO_3_^−^ (μM)*	NO_2_^−^ (μM)*	NH_4_^+^ (μM)*	Chl *a* (μg l^−1^)*
					Initial volume (l)	Final volume (ml)				
S3	65°52.6′N 29°56.5′W	0.6	33.0	BPLR	100	730	137	0.408	2.895	0.133	0.29	1.0
S6	59°20.9′N 29°59.9′W	10.9	35.0	ARCT	96	850	113	0.432	5.437	0.21	0.584	1.7
S12	46°44.5′N 30°0.2′W	18.3	35.9	NADR	93	750	124	0.062	0.315	0.051	0.707	~0.2
S19	34°24.8′N 28°28.9′W	24.1	36.6	NAST	95	500	186	0.011	0.022	0.015	0.169	~0.1

* Data from Gomez-Pereira et al. (2010).

At each station, four sets of substrate incubations were prepared: duplicate live incubations (15 ml each) and single killed control incubations (10 ml seawater + 3.5 ml formalin) for each substrate. Each substrate was added at a final concentration of 3.5 μM monomer-equivalent to the incubations. Incubations from S3 and S6 were incubated at 4°C in the dark in a temperature-controlled room; incubations from S12 and S19 were initially incubated at 18–23°C (in flowing seawater in the lab) and then were incubated at 20°C (temperature-controlled room) in the dark. Subsamples (ca. 2.5 ml) were removed from each incubation at 0, 5, and 15 days, filtered through a sterile 0.2-μm pore-size filter, the first 1 ml of filtrate was discarded, and the remaining 1.5 ml of the filtrate was stored frozen until analysis. In brief, substrate hydrolysis was determined from the changes in polysaccharide molecular weight with time, as measured using gel permeation chromatography and fluorescence detection, described in detail in Arnosti (2003). Gel columns consisted of a 20 cm × 1 cm column of Sephadex G-50 gel, connected in series to a 18.5 cm × 1 cm column of Sephadex G-75 gel. Mobile phase (phosphate buffer, pH 8.0) was pumped at 1 ml min^−1^ by a Shimadzu LC-10AT pump; the column outflow passed through a Hitachi L-7480 fluorescence detector, set to excitation and emission wavelengths of 490 and 530 nm, respectively. Hydrolysis rates were calculated from differences in substrate molecular weight at the different time points, as described in detail in Arnosti (2003).

### FLUORESCENCE *IN SITU* HYBRIDIZATION AND CELL COUNTS

At the end of the 15-day incubation, the remaining incubation water (ca. 7.5 ml) from one of the two live replicates was fixed with particle-free formaldehyde, filtered through polycarbonate 0.2-μm pore-sized filters, and the filters were stored frozen (−20°C) until analysis. *In situ* identification with the standard catalyzed reporter deposition (CARD) FISH protocol (Pernthaler et al., 2002) and cell counting relative to total cell counts (DAPI) was carried out using the general bacterial probe mix EUB338 I–III (Daims et al., 1999) as well as the group-specific probes CF319a (Manz et al., 1996) and PLA46 (Neef et al., 1998; for a recent update of probe specificity see Amann and Fuchs, 2008) targeting *Bacteroidetes* and *Planctomycetales*, respectively. For two samples, insufficient volume was obtained to make accurate FISH counts, so relative abundances were estimated.

## RESULTS

### ENZYMATIC HYDROLYSIS RATES AND PATTERNS

**FIGURE 1:**
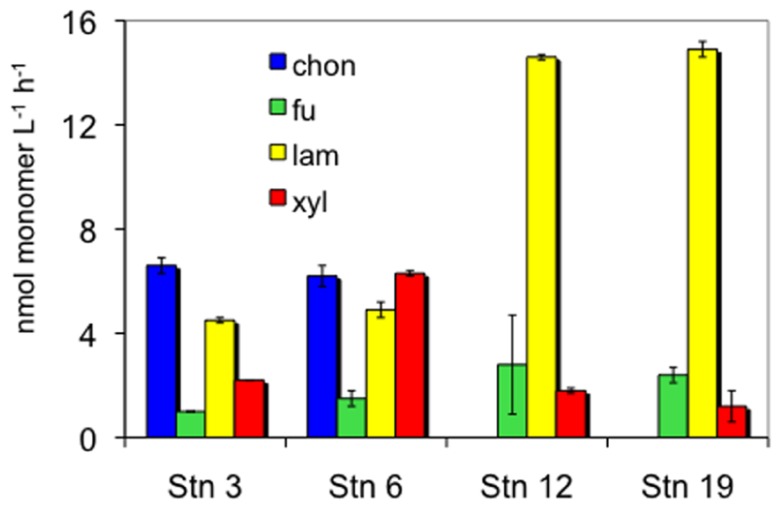
**Enzymatic hydrolysis rates of four polysaccharides in 10 μm retentate from each station**. Chon, chondroitin sulfate; fu, fucoidan; lam, laminarin; xyl, xylan. Bars show standard deviation of two replicate incubations. Hydrolysis rates are maximum rates that were measured after 5 days incubation for all substrates except chondroitin, laminarin at S6, and xylan at S3 and S6, which were from 15 days incubation (see text).

The four stations sampled represent different North Atlantic provinces, as characterized by distinctive temperature and salinity signatures, as well as nutrient and chlorophyll concentrations (**Table 1**; see Gomez-Pereira et al., 2010 for further details). They also showed distinct patterns and rates of extracellular enzymatic activities. **Figure 1** shows the maximum hydrolysis rate of each substrate at each station, which was measured after either 5 or 15 days incubation. Maximal rates were observed after 5 days of incubation for fucoidan from all stations, for laminarin from all stations except S6, and for xylan from S12 and S19. The maximal hydrolysis rates of xylan from S3 and S6, of laminarin from S6, and of chondroitin at S3 and S6 were observed after 15 days of incubation. Data from both time points (5 and 15 days) are plotted in **Figure 1** because in cases where substrates are hydrolyzed rapidly (e.g., maximum values at 5 days), later time points (e.g., 15 days) typically show a lower calculated hydrolysis rate, reflecting the fact that hydrolysis was nearly complete at the earlier time.

The differences in timescales over which hydrolysis rates reach a maximum rate reflect the activity and distribution of extracellular enzymes within a given community. Since hydrolysis of fluorescently labeled polysaccharides is detected as a change in the molecular weight distribution of the entire pool of added substrate (see Arnosti, 1995, 2003 for more details), extended incubations (timescales of days) are typically required to measure activity in pelagic samples. An enzyme activity that is low or is uncommon among a community is first observable after a more extended period of incubation, while enzyme activities that are intrinsically rapid or are widespread among a given community may be observed at an earlier time point. Since these timescales of measurement (days) allow sufficient time for community growth as well as enzyme expression, enzyme activities measured using this technique represent the potential of a community to react to substrate input – e.g., by induction of genes and/or by growth of a numerically small or slow-growing members of the community – rather than reflecting only the activity of enzymes already present at the time substrate is added to the sample. We note also that in this incubation, the time points were quite widely spaced; maximum hydrolysis rates might have been reached prior to 5 or 15 days, so the rates reported here could be underestimated. We also note that these measurements necessarily represent the potential of the microbial community present in the sample at the time of collection. Samples collected at different seasons could show different patterns, depending on larger-scale factors that could drive seasonal variation in microbial community composition.

### HYDROLYSIS PATTERNS BY STATION

As a pair, S3 and S6 resembled each other in hydrolysis rates and patterns. At these stations, all four substrates were hydrolyzed by the large-particle-associated microbial fraction. Hydrolysis rates of chondroitin were quite high; the hydrolysis rates of the other substrates were for the most part within a factor of ca. 1–4 of the chondroitin hydrolysis rate. At S12 and S19, in contrast, hydrolytic activity was highest for laminarin, which was very rapidly hydrolyzed (14.6 and 14.9 nmol l^−1^ h^−1^, respectively). Hydrolysis rates of fucoidan and xylan at S12 and S19 were 5–20 times lower than that of laminarin, and there was no indication of chondroitin hydrolysis in the large-particle-associated fraction from these stations.

### MICROBIAL COMMUNITY COMPOSITION

The composition of the microbial communities at the conclusion of the incubation was investigated using CARD-FISH. The general bacterial probe mixture EUB338 I–III hybridized to 75–99% of all DAPI-stained cells. Of these bacteria, a substantial proportion was assigned to *Bacteroidetes* with probe CF319a: on average, 26, 22, 15, and 17% for S3, S6, S12, and S19, respectively (**Table 2**). Counts with the probe PLA46 specific for *Planctomycetes* were low or below the detection limit at all stations (data not shown), serving also as a negative control for unspecific staining and autofluorescence. For any given station, there was no consistent relationship between hydrolysis rate of a specific substrate and fraction of cells detected by CF319a.

**Table 2:** CF319a hybridized cells, as % DAPI-stained cells, after 15-day incubation with substrate.

**Substrate**	**Station**						
	**S3**	**S6**	**S12**	**S19**
Lam	23.6	13.5	19.4	10–20*
Xyl	28.3	22.7	13.9	5–10*
Fu	17.3	13.9	10.2	19.3
Chon	34.3	36.7	15.8	14.7
*Average*	*25.9*	*21.7*	*14.8*	*17.0*
Bulk water**	19.7	17.5	30.6	5.4

*Lam, laminarin; xyl, xylan; fu, fucoidan; chon, chondroitin sulfate.*

* Estimated abundance: volume too low to count precisely (see text). These values not included in the average.

** Bulk water: CF319a-stained cells (as % of DAPI-stained cells) from a depth of 20 m at each station; data from Gomez-Pereira et al. (2010).

## DISCUSSION

The enzymatic complement of heterotrophic bacteria is variable even among closely related organisms (Gao et al., 2003; Gomez-Pereira et al., 2012), so the nature and type of substrate that can be accessed by a given organism is quite specific, as has been demonstrated by microbiological and genomic investigations of a variety of prokaryotes (e.g., Martinez et al., 1996; Ensor et al., 1999; Glöckner et al., 2003; Bauer et al., 2006; Weiner et al., 2008). Some bacteria are able to use low molecular weight hydrolysis products although they cannot effectively hydrolyze the initial substrates (Cotta, 1992), demonstrating the necessity for close interactions among specific bacteria within a community. The extent to which substrate preference patterns extend from specific organisms to entire microbial communities, however, is only beginning to be explored in marine environments. Previous studies of polysaccharide hydrolysis by pelagic microbial communities demonstrate site-specific differences in extracellular enzymatic hydrolysis rates and patterns (Arnosti et al., 2005a; Steen et al., 2008) as well as large-scale latitudinal gradients in the spectrum of polysaccharide hydrolases activities in surface ocean waters (Arnosti et al., 2011). The observation that enzymatic hydrolysis patterns measured here clustered into two groups, consisting of the northern BPLR/ARCT stations S3 and S6, and the more temperate NADR/NAST stations S12 and S19 (**Figure 1**) suggests that there are biogeographic patterns in enzymatic capabilities also among the large-particle-associated fraction of microbial communities.

**FIGURE 2:**
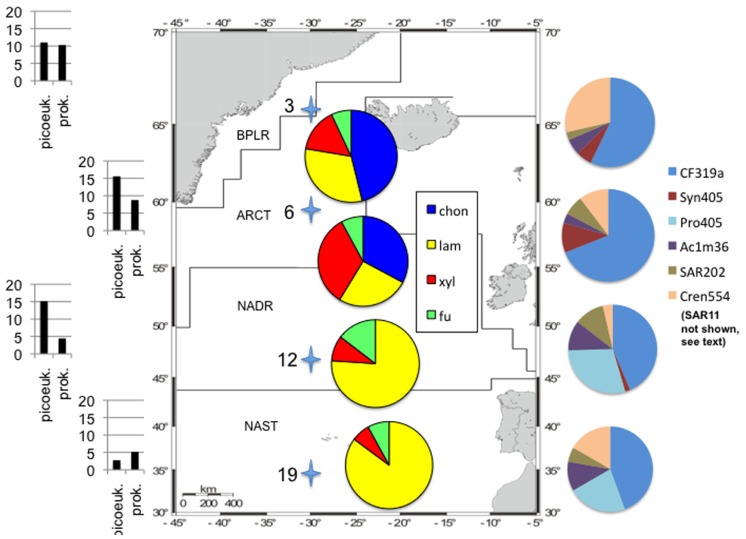
**Study area with oceanic provinces, sampling stations, enzymatic hydrolysis patterns, cell counts, and community composition information**. Station locations shown as stars (station numbers on left); map modified from Gomez-Pereira et al. (2010). Cell counts on left side of figure: total prokaryotic cell counts as “prok,” picoeukaryotes counted via flow cytometry as “picoeuk.” Left hand scale: for picoeukaryotes, ×10^3^ cells ml^−1^, for prokaryotes, ×10^5^ cell ml^−1^. Data from Schattenhofer et al. (2011) and Gomez-Pereira et al. (2010). Pie charts superimposed on map: relative contribution of each enzyme activity to total activity measured at a station; substrate names as in **Figure 1**. Pie charts on right hand side: relative contribution of FISH-stained cells to total cell counts from water samples that were not subject to flow cytometry (“unsorted cells”); data from Schattenhofer et al. (2011) SI **Table 2**. Note that SAR11 cells contributed 25–32% of total DAPI-stained cells at each station, and are not plotted here for clarity. Pie charts as shown represent 17.5, 29, 27.5, and 18% of the total DAPI-stainable cells at S3, S6, S9, and S19, respectively. CF319a, *Bacteroidetes*; SYN405, *Synechococcus*; PRO405, *Prochlorococcus*; AC1M36, marine clade I *Actinobacteria*; SAR202, SAR 202 clade; CREN554, *Crenarchaeota* marine group I.

These functionally distinct communities also differed in composition, as shown by FISH staining of whole water (unsorted) as well as flow cytometry-sorted samples collected concurrently with the present study. Although members of the SAR11 were the single largest constituents of the unsorted population, contributing 25–32% of the total stained cells at a depth of 20 m at all four stations, the contribution of other prokaryotic cells was quite variable by station (Schattenhofer et al., 2011; **Figure 2**). CF319a-stainable cells (*Bacteroidetes*) contributed a large fraction of the identifiable cells, Syn405 (*Synechococcus*) counts were significant only in S3 and S6, while Pro405 (*Prochlorococcus*) counts were significant only at S12 and S19 (**Figure 2**; Schattenhofer et al., 2011). The fundamental factors shaping these differences in microbial community composition remain to be determined (Hanson et al., 2012); as noted by Schattenhofer et al. (2011), specific groups of bacterioplankton were statistically correlated with physical, chemical, and biological parameters, in agreement with investigations of other microbial communities at other locations (Kan et al., 2006; Gilbert et al., 2012). In any case, the general trend of a north to south decrease in nutrient and chlorophyll *a* concentrations as well as picoeukaryotic abundance and prokaryotic counts at S3, S6, S12, and S19 tracks the changes in prokaryotic community composition and extracellular enzyme activities measured at the four stations (**Table 1**; **Figure 2**).

Compositional distinctions among the microbial communities at these four stations were supported also by more detailed analysis of bacteroidetal communities, carried out on the filtrate that had passed through the 10 μm cartridge. A focus on members of the class Flavobacteria of the phylum *Bacteroidetes* is especially relevant due to their proven abilities to degrade organic macromolecules in marine waters (Kirchman, 2002; Bauer et al., 2006), as well as their abundance at these stations and in our incubations (**Table 2**). Analysis of 16S rRNA gene clone libraries and direct cell counts via CARD-FISH demonstrated that the flavobacterial composition of S3 and S6 had considerable overlap, sharing 25–40% of flavobacterial phylotypes, while S3 and S18 were mostly unrelated, sharing just 2–5% of flavobacterial phylotypes (Gomez-Pereira et al., 2010). Flavobacteria are a major target of FISH probe CF319a, which comprised a substantial proportion (5–31%) of cells in bulk seawater (Gomez-Pereira et al., 2010) as well as 15–26% of the post-incubation large-particle-associated fraction from these stations (**Table 2**). Since the complement of glycosyl hydrolase genes in fully sequenced members of the *Bacteroidetes* differs substantially (Gomez-Pereira et al., 2012), these differences in flavobacterial community composition indicate the potential for functional differences.

Evidence of biogeographic patterns in polysaccharide hydrolase activities (**Figures 1 and 2**) is also supported by the results of a metagenomic investigation based on analysis of *Bacteroidetes*-associated fosmids obtained from S3 and S18, the same samples Gomez-Pereira et al. (2010) used for clone libraries and CARD-FISH staining. Fifteen glycosyl hydrolase-associated genes were identified in the fosmids, some of which included signal sequences that predicted enzyme export to the outer membrane (Gomez-Pereira et al., 2012), where they could hydrolyze extracellular polysaccharides. At S3, genes corresponding to sulfatases (used to remove sulfate groups from polysaccharides) were also identified; most of these enzymes were likewise predicted to be exported from the cytoplasm (Gomez-Pereira et al., 2012), and thus to play a role in extracellular hydrolysis. Sulfatase genes were also identified at S18, in lower numbers than at S3. Overall, the relative content of glycosyl hydrolases and sulfatases at S3 was higher than at S18; differences in gene abundance were correlated with the abundance of the organisms from which the fosmid sequences were derived (Gomez-Pereira et al., 2012). The current study thus provides evidence of functional differences that are implied by the genomic and community population data from samples collected concurrently from these same stations (Gomez-Pereira et al., 2010, 2012; Schattenhofer et al., 2011).

Although differences in patterns of enzymatic hydrolysis (**Figures 1 and 2**) suggest fundamental differences in function of the large-particle-associated fraction of the community, information about relative gene abundance at different stations (Gomez-Pereira et al., 2012) cannot be linked directly to specific rates of hydrolysis. The extent and conditions under which a given gene is expressed in the environment are unknown; moreover, the kinetic characteristics of the enzymes themselves as well as the quantity of enzymes produced contribute to observed hydrolysis rates. Furthermore, the hydrolysis rates measured in this study may be affected by the fraction of large particles retained by the 10 μm cartridge filter, as well as by the extent of particle colonization by bacteria at each station. The DNA extractions of Gomez-Pereira et al. (2010), 2012 were carried out on the fraction of water that passed through the 10 μm cartridge, i.e., on the filtrate, rather than the retentate, and thus specifically excluded the large-particle-associated fraction used to measure enzyme activities in the current study. The retentate fraction, however, was isolated within a background of retained seawater (i.e., particles were not isolated from their surrounding solution), and therefore also contained some cells that were present in the last fraction to pass into the cartridge filter. Substrate hydrolysis rates showed no systematic relationship with concentration factor (**Table 1**), also indicating that the differences observed among stations were not an outcome of sample manipulation. Furthermore, Schattenhofer et al. (2011) did not prefilter their samples, and the population they studied (**Figure 2**) would thus also include the fraction defined here as large-particle-associated bacteria. In any case, any differences between stations in bacterial cell numbers of the large-particle-associated fraction would be reflected primarily in differences in the rates of hydrolysis, and not in the patterns of substrates hydrolyzed. Different patterns of substrate hydrolysis point instead at functionally different communities.

The patterns evident at these stations additionally demonstrate that enzymatic hydrolysis rates in pelagic waters are not a simple function of environmental temperature. Although *in situ *as well as incubation temperatures at S12 and S19 were substantially warmer than at S3 and S6, only laminarin hydrolysis rates appear to track temperature. Hydrolysis rates of fucoidan, chondroitin, and xylan, in contrast, were comparable to or higher at the colder stations (S3/S6) than at the warmer stations (S12/S19). A correlation of laminarin hydrolysis with temperature, and little temperature correlation for other polysaccharides, is in fact a pattern consistent with enzyme activities in surface ocean waters on latitudinal gradients (Arnosti et al., 2011).

The lack of measurable chondroitin hydrolysis at S12 and S19 is particularly interesting in light of the observation that it is one of the activities frequently measured in surface ocean waters (Arnosti et al., 2011). At these stations, hydrolytic activity may be associated with the free living or small-particle-associated microbial fraction that would not have been retained by a 10-μm filter. This activity may also simply be missing in this biogeographic province, since evidence to date suggests that enzymatic capabilities are non-uniformly distributed in the surface ocean (Arnosti et al., 2005a, 2011; Steen et al., 2008), and polysaccharide hydrolase gene distribution varied among these stations (Gomez-Pereira et al., 2012). Conversely, fucoidan hydrolysis at all four stations is also notable, since it is an activity that is relatively infrequently measured in surface ocean waters (Arnosti et al., 2011), and perhaps is primarily associated with large-particle-associated bacteria. Both chondroitin and fucoidan are sulfated polysaccharides, and their rates and extent of utilization may also be related to the activities of sulfatase enzymes whose genes were identified in the fosmids from these stations (Gomez-Pereira et al., 2012). The pattern of more rapid chondroitin hydrolysis upon extended incubation (i.e., 15 days) is consistent with previous observations of chondroitin hydrolysis in marine waters and sediments, as well as experiments indicating that chondroitin hydrolysis is induced in marine bacteria (Arnosti, 2004).

Our CARD-FISH identifications focused on the potential enrichment of two bacterial phyla that had been linked to degradation of (sulfated) polysaccharide, *Planctomycetales* (Glöckner et al., 2003; Woebken et al., 2007) and *Bacteroidetes* (Kirchman, 2002; Bauer et al., 2006). Whereas we could not detect significant numbers of *Planctomycetales* with probe PLA46, there was consistently a strong contribution of CF319a-positive bacteroidetal cells in the large-particle-associated fraction post-incubation (**Table 2**). This result is consistent with the observation that members of the phylum *Bacteroidetes* – and in particular those of the class *Flavobacteria* – are frequently associated with phytoplankton blooms (Gomez-Pereira et al., 2012; Teeling et al., 2012). FISH staining showed somewhat higher average counts of CF319a-stainable cells at S3/S6 compared to S12/S19, a result likely due to initial differences in phytoplankton (**Figure 2**; Gomez-Pereira et al., 2010) as well as to microbial growth in response to substrate addition. Specific differences in functional potential of these organisms, however, became evident only through direct measurement of extracellular enzyme activities, using an experimental approach that provides information about structural specificities of these enzymes. This investigation is the first to specifically focus on the hydrolytic capabilities of large-particle-associated bacteria to hydrolyze these substrates; it is also the first investigation to combine FISH staining directly with these measurements. These communities evidently exhibit distinct patterns in enzyme activities, as has been observed for unfiltered surface and subsurface waters (Arnosti et al., 2005a, 2011; Steen et al., 2008, 2012). The extent to which patterns of enzyme activities may differ for large particles vs. whole water should be a focal point of further work, as should the extent to which such patterns may change through annual cycles of phytoplankton and bacterial succession (Allison et al., 2012; Gilbert et al., 2012). Such investigations will help us define more precisely at a functional level the contributions of specific microbial communities to carbon processing in the ocean.

## Conflict of Interest Statement

The authors declare that the research was conducted in the absence of any commercial or financial relationships that could be construed as a potential conflict of interest.
